# Postoperative Deep Vein Thrombosis in Patients Undergoing Surgery With Curative Intent for Colorectal Cancer: A Prospective Cohort Study

**DOI:** 10.7759/cureus.93316

**Published:** 2025-09-26

**Authors:** Neerav D Aruldas, Albert Kota, Deepa R Korula, Geethu E Punnen, Mark R Jesudason

**Affiliations:** 1 General and Colorectal Surgery, Christian Medical College Vellore, Vellore, IND; 2 Vascular Surgery, Flinders Medical Centre, Adelaide, AUS; 3 Radiology, Madha Medical College, Chennai, IND; 4 Radiology, Bedfordshire Hospitals NHS Foundation Trust, Bedford, GBR

**Keywords:** colon cancer, colorectal cancer, deep vein thrombosis (dvt), low-molecular-weight heparin, rectal cancer, thromboprophylaxis, venous thromboembolism

## Abstract

Background

Deep vein thrombosis (DVT) is a known postoperative complication associated with morbidity and mortality. Colorectal cancer patients have a higher risk of developing postoperative DVT, considering the long duration of surgery, positioning during surgery, pelvic dissection, and neoadjuvant chemoradiotherapy. For them, current guidelines recommend a protocol of 28-day postoperative pharmacological thromboprophylaxis with low-molecular-weight heparin (LMWH), unfractionated heparin, or fondaparinux, along with mechanical thromboprophylaxis like graduated compression stockings (GCS). However, most patients receive only in-hospital thromboprophylaxis. The aim of this study was to estimate the frequency of DVT in patients undergoing curative intent surgery for colorectal cancer, followed by postoperative thromboprophylaxis with GCS during their in-hospital stay in the Indian population.

Methods

Patients undergoing curative intent surgery for colorectal cancer were screened for occult pre-operative DVT including a duplex ultrasound. All patients received in-hospital thromboprophylaxis with LMWH and GCS and were followed up for 28 days to six months after surgery to screen for symptomatic and asymptomatic DVT during their follow-up.

Results

Of the 42 patients recruited to the study, 13 were followed up with Doppler ultrasonography and 29 were followed for symptoms of DVT telephonically. No asymptomatic DVT or symptomatic DVT was detected during the follow-up. There was no significant difference in the risk factors between those who underwent follow-up Doppler ultrasonography and those who did not, except that those who did have follow-up Doppler ultrasonography had a greater exposure to neoadjuvant and/or adjuvant therapy.

Conclusions

Our study suggests that thromboprophylaxis during the in-hospital postoperative period along with GCS was sufficient for preventing symptomatic or asymptomatic postoperative DVT in patients undergoing curative-intent surgery for colorectal cancer. However, a study with a larger sample size is required to recommend this for thromboprophylaxis in patients undergoing surgery for colorectal cancer in India.

## Introduction

Deep vein thrombosis (DVT) is a well-known postoperative complication, particularly among patients with cancer [1,2]. DVT is a significant cause of morbidity and mortality among patients with colorectal cancers [3,4] due to the increased risk associated with undergoing pelvic surgery of long duration, positioning during surgery, comorbid conditions, and neoadjuvant and adjuvant therapies. In addition, due to other risk factors, the colorectal cancer patients are categorized as high risk for developing postoperative DVT with a score of five or more when assessed using the Caprini risk assessment model [5,6].

To prevent postoperative DVT, several international guidelines, such as Enhanced Recovery After Surgery (ERAS) Society, the National Institute for Health and Care Excellence (NICE), the American Society of Colorectal Surgeons (ASCRS), and the American College of Chest Physicians (ACCP), have recommended postoperative DVT prophylaxis in the form of pharmacological as well as mechanical prophylaxis. The Caprini risk assessment model for venous thromboembolism (VTE) recommends a postoperative protocol of pharmacological thromboprophylaxis with low-molecular-weight heparin (LMWH), unfractionated heparin (UFH) or fondaparinux along with mechanical thromboprophylaxis like graduated compression stockings (GCS) for high-risk patients [7-10]. The recommended durations of thromboprophylaxis as per these guidelines are (i) standard duration of at least seven days for major abdominal surgeries; (ii) extended duration of 28 days for patients undergoing major open or laparoscopic abdominal or pelvic surgery for cancer including surgery for colorectal cancer; and (iii) duration of in-hospital stay recommended only for patients with a Caprini Risk Score of up to four.

Studies have indicated that there is a lower reported incidence of DVT/VTE in Asian populations [11,12]. A retrospective study over a 10-year period conducted in India among all patients diagnosed with VTE between 1996 and 2005 showed a VTE incidence of 17.46 per 10,000 admissions, and those following surgery were five per 10,000 surgeries, most of them (40.3%) being after general surgery [13]. A multicentric observational study conducted in India among 298 cancer patients who underwent abdominal or pelvic surgery found that none of the patients had symptoms of DVT [14]. However, duplex ultrasound studies were not done on any of them, and the duration of thromboprophylaxis given varied across centers.

The reported incidence of postoperative DVT in colorectal cancer patients ranges from 3.3% to 17.7% [1,4,15-18]. This risk remains high up to six months after surgery [18]. Studies have shown a reduction in VTE and its consequences following the extended thromboprophylaxis regimen [15,19-21]. However, other studies [22-24] have shown that only 8-27% of surgeons followed the recommended 28-day pharmacological thromboprophylaxis after colorectal cancer surgery. In a national survey, the majority (68%) of surgeons reported that they started thromboprophylaxis on admission and stopped it at discharge [23].

There is limited data on the incidence of postoperative DVT among colorectal cancer patients in the Indian context. A randomized study conducted in India with 99 patients who underwent curative surgery for colorectal cancer showed no instances of DVT on the 6 ± 1 postoperative day as confirmed by Doppler ultrasonography, regardless of whether the patients received LMWH prophylaxis (n=51) or not (n=48) [25]. The aim of our study was to estimate the frequency of postoperative DVT in Indian patients with colorectal cancer who have undergone curative intent surgery followed by postoperative thromboprophylaxis along with GCS during in-hospital stay.

## Materials and methods

Study design and setting

A prospective observational cohort study was conducted from February 2019 to September 2020 by the colorectal unit of a tertiary care teaching institute (Christian Medical College, Vellore) in the state of Tamil Nadu, in Southern India. The study protocol for patients undergoing curative-intent surgeries for colorectal cancer included (a) risk assessment for developing postoperative DVT using the Caprini risk assessment model [5,6], (b) pre-operative assessment of DVT by a duplex ultrasound, performed by a consultant radiologist, to establish baseline status of the deep venous system as well as to identify patients with pre-existing DVT. Doppler ultrasonography was of both lower limbs and internal deep veins (the inferior vena cava, and the internal, external, and common iliac veins) to check for compressibility, phasicity, augmentation and flow in both lower limbs, and to assess flow in the internal deep veins, (c) one dose of LMWH or UFH at 6 PM on the evening prior to the surgery to all patients, (d) postoperative thromboprophylaxis with LMWH or UFH with GCS during the hospitalization period for all patients, (e) neoadjuvant and adjuvant chemo and radiotherapy was based on the TNM staging, the depth of penetration (T stage), involvement of lymph nodes (N stage) and the presence or absence of distant metastasis (M stage), as per the American Joint Committee on Cancer (AJCC) classification [26] (f) postoperative assessment of DVT in one to six months by a duplex ultrasonography doppler study done by a consultant radiologists who did the pre-operative assessment. Both open and laparoscopic surgeries were performed by the colorectal unit.

Inclusion and exclusion criteria

Inclusion Criteria

Inclusion criteria include: (i) Age ≥18 years; (ii) Diagnosis of colon or rectal cancer; (iii) Undergoing curative-intent surgery in the institute’s colorectal unit.

Exclusion Criteria

Exclusion criteria are as follows: (i) Previously diagnosed DVTA and (ii) symptomatic DVT detected on pre-operative duplex ultrasonography screening.

Caprini risk assessment model

The Caprini risk assessment model is a validated method of assessing the risk of development of VTE (Table 1) [5]. The Caprini Risk Score, the sum of the points based on the risk factors present, determines the risk levels and is categorized as low risk (score of 0-1), moderate risk (score of 2), high risk (score of 3-4), and highest risk (score of >5).

**Table 1:** Scoring for risk factors in Caprini risk assessment model

Score 1 point for each risk factor	Score 2 points for each risk factor	Score 3 points for each risk factor	Score 5 points for each risk factor
Age 40-60 years	Age 61-74 years	Age >75 years	Stroke (<1 month)
Minor surgery	Arthroscopic surgery	History of VTE	Elective arthroplasty
BMI >25 kg/m^2^	Major open survey (>45 minutes)	Family history of VTE	Hip, pelvis, or leg fracture
History of major surgery (<1 month)	Laparoscopic surgery (>45 minutes)	Positive factor V Leiden	Multiple trauma (<1 month)
Varicose veins	Cancer (past or present)	Positive prothrombin 20210A	Acute spinal cord injury (<1 month)
Swollen legs	Patient confined to bed (>72 hours)	Elevated serum homocysteine	
Acute myocardial infarction	Immobilizing plaster cast (<1 month)	Positive lupus anticoagulant	
Congestive heart failure (<1 month)	Central venous access	Elevated anticardiolipin antibodies	
Sepsis (<1 month)		Heparin-induced thrombocytopenia	
Serious lung disease such as pneumonia (<1 month)		Other congenital or acquired thrombophilia	
Chronic obstructive pulmonary disease			
Medical patient on bed rest			

Postoperative follow-up

The patients were asked to return to the outpatient services of the unit for a follow-up with duplex ultrasound to investigate for asymptomatic DVT 28 days to six months after surgery. Those who could not return to the outpatient services were followed up with tele-consultation to assess if they had any symptoms of DVT.

Data collection

A survey questionnaire was developed to collect data on patients’ background characteristics, risk factors such as body mass index (BMI) and co-morbidities, clinical and radiological findings, operative and postoperative details along with complications, if any, and follow-up data 28 days after surgery. The information was collected by interviewing the patients and from their hospital records.

Statistical analysis

The data were processed and analysed using STATA version 18.0 (STATA Corporation, College Station, TX, USA). The primary outcome was the development of postoperative DVT. Descriptive analysis of frequencies (mean, median, and standard deviation (SD)) and percentages of patients’ characteristics such as age, sex, body mass index (BMI), co-morbidities, and other risk factors, and Caprini Risk Score were carried out. Bivariate analysis of postoperative complications and duration of surgery, with the type of surgery performed, was carried out. Fisher's exact test and the Mann-Whitney U test were performed to determine associations of risk factors for developing postoperative DVT among those who had and did not have a postoperative Doppler sonography and were considered significant at a p-value of <0.05.

Ethical considerations

Before the commencement of the study, the study was reviewed and approved by the Institutional Review Board at Christian Medical College, Vellore, India (IRB Min. No.11710 [OBSERVE] dated 03-12-2018). The consent form was prepared as per the institutional guidelines and translated into Tamil, Hindi, Telugu, and Bengali. Written informed consent was obtained from patients undergoing curative-intent surgery for colorectal cancer for participation in the study.

## Results

Demographic characteristics, comorbidities, and risk factors

Between February 2019 and September 2020, 43 patients were recruited for the study. One patient was excluded from the study as his preoperative DVT screening had shown asymptomatic DVT. Therefore, 42 patients were included in the analysis. Most of them were males (78.6%), were between the ages of 20 - 74 years, with a mean age of 47.3 years (SD:13.04) and a median age of 48.5 years (Table 2). None of them had a family history of DVT or pulmonary embolism; 16 (38.1%) were smokers, chewed tobacco, or had alcohol, and 14 (33.0%) were obese (BMI>25kg/m^2^). Overall, 19 patients (45.2%) had one or more comorbidities, and hypertension in 12 patients (28.6%) and diabetes in 10 patients (23.8%) were the main comorbidities.

**Table 2:** Demographic characteristics, comorbidities, and other risk factors

Categories	Results (N=42)	
	n	%
Age		
20 years	1	2.4
21-40 years	10	23.8
41-60 years	25	59.5
61-74 years	6	14.3
Sex		
Male	33	78.6
Female	9	21.4
Body Mass Index		
<25kg/m^2^	28	66.7
>25kg/m^2^	14	33.3
Comorbidities		
Diabetes	10	23.8
Hypertension	12	28.6
Ischaemic heart disease	1	2.4
Cerebrovascular accident	1	2.4
Chronic obstructive pulmonary disease	1	2.4
Hypothyroidism	1	2.4
None	25	59.5
Alcohol or Tobacco exposure		
Smoked or chewed tobacco	14	33.3
Chronic alcohol consumption	2	4.8
None	26	61.9

Cancer site, histopathological grading, TNM staging, and neoadjuvant therapy

A higher number of patients had rectal cancer (59.5%, n=25) than colon cancer (40.5%, n=17). Among the 17 patients who had colon cancer, the most common site was right colon (n=8), and others were synchronous adenocarcinoma of the right and sigmoid colon, metachronous recurrence at the ileo-transverse anastomosis after a right hemicolectomy was done for adenocarcinoma caecum seven years ago and had received three cycles of adjuvant chemotherapy, and multiple malignant colonic polyps in the sigmoid colon, transverse colon, and the hepatic flexure (Table 3). Most of the colorectal patients had moderately differentiated adenocarcinoma (66.7%, n=28), and were in TNM stage IIIB (45.2%, n=19) (Table 3). The Caprini Risk Score ranged from 4-7 (mean: 5.2, SD: 0.79), 19.0% had a score of four, 38.1% had a score of five, 26.2% had a score of six, 4.8% had a score of seven, and 81% (n=34) had a score of 5 or more. Twenty-two out of the 25 patients who had rectal cancer, mostly in TNM stage IIIB, received neoadjuvant chemoradiotherapy. They were operated between four and 30 weeks (mean: 9.6 weeks, SD: 5.84) of neoadjuvant chemoradiotherapy.

**Table 3:** Distribution of site of cancer, histopathology, and TNM staging

Histopathological distribution	Total (N=42)	
	n	%
Site of colorectal cancer		
Rectum	25	59.5
Right colon	8	19.0
Transverse colon	2	4.8
Left colon	2	4.8
Sigmoid/ Rectosigmoid	3	7.1
Synchronous right and sigmoid colon	1	2.4
Multiple malignant colonic polyps	1	2.4
Histopathology		
Well-differentiated adenocarcinoma	2	4.8
Moderately differentiated adenocarcinoma	28	66.7
Poorly differentiated adenocarcinoma	4	9.5
Unclassified adenocarcinoma	3	7.1
Diffuse large B-cell lymphoma	1	2.4
Tubulovillous adenoma with high-grade dysplasia	3	7.1
Not available	1	2.4
TNM stage		
Stage I	5	11.9
Stage IIA	5	11.9
Stage IIB	3	7.1
Stage IIC	1	2.4
Stage IIIA	1	2.4
Stage IIIB	19	45.2
Stage IIIC	2	4.8
Not available	2	4.8

Surgical details and follow-up

Twenty-three (54.8%) surgeries were open procedures, two of which were started laparoscopically but were converted to open procedures, and 19 (45.2%) were laparoscopic surgeries. Of the 25 patients with rectal cancer, the common procedures were 11 (44%) abdominoperineal excision and nine (36%) low ultralow anterior resections. Of the 17 colon cancer procedures, the most performed procedure was six (35.3%) right hemicolectomies since the right colon was the most common location of colonic tumors. The duration of surgery available for 26 patients ranged from 60 to 300 minutes (mean: 195 minutes, SD: 57 minutes) for open procedures, and from 180 to 300 minutes (mean: 225 minutes, SD: 51 minutes) for laparoscopic surgeries. Overall, the maximum duration of any type of surgery was 300 minutes.

Postoperative complications occurred in 23 patients (54.8%), including 12 who had open surgery, 10 who had laparoscopic surgery, and one who had conversion from laparoscopy to open surgery. The most common complications following laparoscopic surgery were surgical site infection (n=7) and anastomotic leak (n=3), while the common complications after open procedures were urinary tract infection (n=4), and surgical site infections (n=3). All 42 patients received postoperative thromboprophylaxis along with GCS to prevent DVT. The study protocol included postoperative thromboprophylaxis with GCS during their in-hospital stay, and the duration of thromboprophylaxis was longer in patients with complications (mean: 10.3 days; SD: 5.06; range: 0-21 days) compared to those without complications (mean: 6.2 days; SD: 1.72; range: 1-9 days).

All patients were advised to visit the hospital for a Doppler ultrasonography four to 24 weeks after surgery to detect asymptomatic DVT. Due to travel restrictions during the COVID-19 pandemic, only 13 patients (31.0%) were able to visit the hospital for a Doppler ultrasonography at a mean duration of 9.85 weeks (range: 4-24 weeks) postsurgery and were found to have no DVT. The others who were followed up through teleconsultations for symptomatic DVT did not report any symptoms of DVT.

Among patients who underwent Doppler ultrasonography (n = 13), the mean duration of thromboprophylaxis was 9.08 days (SD: 4.75). Of these, 76.9% (n = 10) had a Caprini risk score ≥5 (mean: 5.08; SD: 0.76); 53.8% (n = 7) had at least one comorbidity; 69.2% (n = 9) experienced postoperative complications; 15.4% (n = 2) had advanced-stage colorectal cancer (Stage IIIA/B/C); and 15.4% (n = 2) had a history of addiction. Fisher’s exact test revealed no statistically significant association between undergoing follow-up doppler ultrasonography and the following factors: Caprini risk score ≥5 (p = 0.6861), presence of comorbidities (p = 0.5159), BMI >25 (p = 0.1587), postoperative complications (p = 0.3166), advanced-stage cancer (p = 0.4525), and addiction history (p = 0.0835). There was also no significant difference in mean thromboprophylaxis duration between those who did and did not undergo Doppler ultrasonography (Mann-Whitney U = -0.7482, p = 0.4533) (see Table 4). However, there was a statistically significant association between undergoing Doppler ultrasonography and receipt of neoadjuvant or adjuvant therapy, or both (p = 0.0414) (see Table 4).

**Table 4:** Differences in risk factors between those who had postoperative Doppler ultrasonography and those who did not *p-value < 0.05 is considered statistically significant

Categories	Postoperative Doppler ultrasonography done			
	Yes	No	Test used	p-value
Caprini Risk Score >5				
Yes	10	24	Fisher's exact test	0.6861
No	3	5		
Comorbidities				
Yes	7	12	Fisher's exact test	0.5159
No	6	17		
Body Mass Index >25				
Yes	2	12	Fisher's exact test	0.1587
No	11	17		
Neoadjuvant or adjuvant therapy or both				
Yes	11	14	Fisher's exact test	0.0414*
No	2	15		
Duration of thromboprophylaxis				
Mean duration (days)	9.08	8.21	Mann-Whitney U test	0.4533
Postoperative complications				
Yes	9	14	Fisher's exact test	0.3166
No	4	15		
Advanced cancer (Stage IIIA/IIIB/IIIC)				
Yes	2	9	Fisher's exact test	0.4525
No	11	20		
Alcohol or tobacco exposure				
Yes	2	14	Fisher's exact test	0.0835
No	11	15		

## Discussion

The aim of this study was to estimate the frequency of postoperative DVT in Indian patients with colorectal cancer who had undergone curative intent surgery and had postoperative pharmacologic plus mechanical thromboprophylaxis for the hospitalization period. One patient had a 30-week gap as the surgery had to be postponed because of COVID-19 infection; all the rest were operated between 4 and 16 weeks of neoadjuvant chemoradiotherapy. The results showed that none of the patients who had duplex ultrasound had asymptomatic DVT, and none of those who followed up through teleconsultation had symptomatic DVT.

For patients undergoing surgeries for colorectal cancer, the current guidelines recommend a 28-day postoperative pharmacological thromboprophylaxis along with mechanical thromboprophylaxis like GCS [7-10]. Choi et al. (2011) [3] and Khorana et al. (2011) [2] showed that patients with cancer are at a higher risk of DVT than those without. RCTs done by Kakkar et al. [15], Vedovati et al. [19] and several other studies have shown the benefits of extended 28-day thromboprophylaxis. A Cochrane review by Rasmussen et al. [27] reviewed randomized controlled trials studying the efficacy of prolonged thromboprophylaxis with LMWH in the prevention of postoperative VTE in patients undergoing abdominal or pelvic surgery. The incidence of 0.2% symptomatic VTE in patients receiving prolonged thromboprophylaxis with LMWH was significantly lower compared to 1.7% in the control group who received in-hospital thromboprophylaxis and either placebo/no-treatment during the extended period. However, other studies [22-24] have shown that only 8-27% of surgeons followed the recommended 28-day pharmacologic plus mechanical thromboprophylaxis after colorectal cancer surgery. In a national survey, the majority (68%) of surgeons reported that they started thrombo-prophylaxis on admission and stopped it at discharge [23].

Choi et al. (2011) [3] showed that the risk increased with age, BMI, the presence of one or more medical comorbidities, the site and stage of the cancer, and the administration of chemotherapy. Our study patients had multiple risk factors: Caprini Risk Score of >5 (81%), one or more comorbidities (46.2%), addictions (59.5%), advanced cancer (Stage IIIA/B/C) (35.5%), long duration surgeries of more than 45 minutes, a high-risk factor included in the Caprini Risk Score, and postoperative complications (69.2%). Comparing the patients who had follow-up duplex ultrasound and those who did not, they had similar risk profiles except that the group who had follow-up DVT had more exposure to neoadjuvant or adjuvant therapy, which increased their risk profile. Though the study patients had high Caprini Risk Scores and multiple risk factors, and though the duration of the postoperative pharmaco-mechanical thromboprophylaxis was only for the in-hospital period, there was no recorded instance of DVT during the follow-up scans. 

Studies have shown that Asian populations [11,12] and some from India [13,14,25] have a lower incidence of DVT in abdominal and pelvic surgeries in general and in colorectal cancers specifically. This may partly explain the absence of DVT in our study patients. Overall, our study indicates that postoperative pharmacological plus mechanical thromboprophylaxis for the in-hospital period was adequate to prevent DVT in Indian patients undergoing curative-intent surgery for colorectal cancer.

Limitations

The major limitations of this study were a small sample size and restricted follow-up Duplex ultrasound, both due to the COVID-19 pandemic travel restrictions. Although no symptoms of DVT were reported among those who were followed up through teleconsultations, their asymptomatic DVT could not be ruled out. As per the Caprini risk assessment model, surgeries lasting more than 45 minutes are considered a risk factor for developing DVT. Although data on the duration of surgery were available for only 26 patients, all surgeries with curative intent for colorectal cancer at our center exceeded 45 minutes in duration. Therefore, the absence of duration data for the remaining cases is unlikely to have affected the assessment of DVT risk.

## Conclusions

Internationally, DVT is a significant cause of postoperative morbidity and mortality, especially among colorectal cancer patients having surgery. The patients included in this study did not have any symptomatic or asymptomatic DVT, though they had multiple risk factors for developing DVT.

While an extended course of pharmaco-mechanical thromboprophylaxis is recommended for patients undergoing surgery with curative intent for colorectal cancer, this study observed that pharmaco-mechanical thromboprophylaxis administered only during the hospital stay was sufficient to prevent postoperative asymptomatic DVT. These findings suggest a potentially lower risk of postoperative DVT in this patient population; however, larger prospective, multicentric studies are required to establish context-specific recommendations for practice in India.

## Appendices

Survey questionnaire

Serial number: Name: Hospital number: Age (years): Sex: M/F Height ht (cm): Weight wt (Kg): BMI: Place of origin: Comorbidities: Diabetes dm Y/N Hypertension htn Y/N Ischemic heart disease ihd Y/N COPD Y/N Previous DVT ph Y/N Family history of DVT fh Y/N Smoking Y/N Alcohol Y/N Site of CRC (1- right/ 2-transverse/ 3-left/ 4-sigmoid/ 5-rectosigmoid/ 6- rectum): Histological group (1- Well Differentiated/ 2-Moderately Differentiated/ 3-Poorly Differentiated/3- Mucinous/ 4-others): Radiological staging 1-C 2- mr

radiological t stage- 1-TX 2- T0. 3-‘Tis. 4-T1 5-T2. 6-T3. 7- T4a. 8- T4b radiological n stage- 1- Nx. 2- N0. 3- N1a. 4- N1b 5- N1c 6- N2a. 7- N2b radiological m stage- 1- M0 2- M1a 3- M1b 4-M1c 5-Mx N

ACT Neoadjuvant Chemotherapy Y/N Duration of NACT. In weeks type of NACT. 1- folfox 2- capeox 3- folfirinox 4- 5fu/luv 5-capecitabine 6- folfiri 7-others Other NACT specify the number of cycles of NACT NART Neoadjuvant Radiotherapy Y/N Duration of NART. IN WEEKS NART Total greys. In gy no of fractions of NART duration bw NACRT and Sx. In weeks preop DVT date. Dd.mm.yyyy preop dvt present Y/n ate of surgery. Dd.mm.yyyy Type of surgery (1- Laparoscopic/2- Open/ 3- lap converted to open/ 4- Transanal excision) Duration of operation in mins adverse events. post op 1- leak 2- bleed 3- others 4- none Duration of thromboprophylaxis. In days

Pathological staging 1-P 2- yp Pathological T stage- 1-TX 2- T0. 3-‘Tis. 4-T1 5-T2. 6-T3. 7- T4a. 8- T4b Pathological n stage- 1- Nx. 2- N0. 3- N1a. 4- N1b 5- N1c 6- N2a. 7- N2b

Pathological m stage- 1- M0 2- M1a 3- M1b 4-M1c 5-Mx post op can done? Y/n post op scan Date Dd.mm.yyyy post op weeks. In weeks post op dvt present. Y/n If DVT screening not done: telephonic interview

 Calf Pain y/n Leg swelling y/n Breathlessness y/n

DVT Screening

**Table 5:** Right side

Preop/Postop	Compressibility		Phasicity		Augmentation		Flow	
IVC								
External iliac veins								
Common Femoral								
Femoral								
Popliteal								
Distal Veins								

**Table 6:** Left side

Preop/Postop	Compressibility		Phasicity		Augmentation		Flow	
IVC								
External iliac veins								
Common Femoral								
Femoral								
Popliteal								
Distal Veins								
